# ABCG2 is a potential prognostic marker of overall survival in patients with clear cell renal cell carcinoma

**DOI:** 10.1186/s12885-017-3224-6

**Published:** 2017-03-27

**Authors:** Haofei Wang, Fangxiu Luo, Zhe Zhu, Zhaoping Xu, Xin Huang, Renyi Ma, Hongchao He, Yu Zhu, Kun Shao, Juping Zhao

**Affiliations:** 1 Department of Urology, Ruijin Hospital, Shanghai JiaoTong University School of Medicine, Building 6th, Floor 6th, 197# Ruijin 2nd road, Shanghai, 200025 China; 2 Ruijin North Hospital, Department of Pathology, Shanghai JiaoTong University School of Medicine, Shanghai, 201801 China; 3 Department of Stem Cell Biology and Regenerative Medicine, Cleveland Clinic, Lerner Research Institute, Cleveland, OH 44195 USA

**Keywords:** ABCG2, ATP-binding-cassette transporters, Biomarker, Overall survival, Renal cancer

## Abstract

**Background:**

ATP-binding cassette sub-family G member 2 **(**ABCG2) is a semi-transport protein that plays a major role in multidrug resistance. We aimed to evaluate the prognostic significance of ABCG2 expression in patients with clear cell renal cell carcinoma.

**Methods:**

From 2008 to 2013, 120 patients with clear cell kidney cancer underwent surgery with paraffin-embedded specimens and necessary clinical information available. Immunohistochemistry staining was performed to grade the expression of ABCG2 as ABCG2(−): less than 10% of tumor cells stained; ABCG2(+): weak membrane staining; and ABCG2(++): moderate or strong membrane staining. The overall survival was analyzed using Kaplan-Meier method. Multivariable Cox regression evaluated the independent predictors for overall survival.

**Results:**

ABCG2(−) was diagnosed in 57 (48%) patients, ABCG2(+) in 52 (43%) patients, and ABCG2 (++) in 11(9.2%) patients. ABCG2 expression significantly correlated with the five-year survival (*p* < 0.001) and distant metastasis (*p* = 0.001). In the multivariable analysis, besides Fuhrman grade, the ABCG2 expression was an independent prognostic marker for overall survival (*p* < 0.001) when incorporating other relevant tumor and clinical parameters (HR = 3.84, 95% CI: 1.92–7.70).

**Conclusion:**

The current data suggests that ABCG2 may serve as a prognostic marker for overall survival in patients with clear cell renal cell carcinoma. Further studies with large cohorts of patients will be essential for validating these findings and defining the clinical utility of ABCG2 in the patient population.

**Electronic supplementary material:**

The online version of this article (doi:10.1186/s12885-017-3224-6) contains supplementary material, which is available to authorized users.

## Background

Renal cell carcinoma (RCC) is the most common type of malignant renal cancer in adults, responsible for approximately 90–95% of the diagnosed cases [1]. Approximatelys, 25–30% patients present metastasis at the time of diagnosis and 30% of the patients relapse after renal surgery [2]. To date, surgery is the primary treatment for RCC, and the five-year survival rate is 65–90%; however, the outcome is considerably reduced in metastatic cases [3]. RCC is relatively resistant to radiotherapy and chemotherapy with only a 4–5% response rate [4, 5]. Some cases respond to immunotherapy with a 30% response rate [6]. By 2013, with the advancement in targeted therapy, such as Sunitinib and Sorafenib, the average survival time was improved from 12 months to 22 months in patients with metastatic RCC [7, 8]. However, the five-year overall survival for metastatic RCC remains <10% [3].

ATP-binding cassette sub-family G member 2 (ABCG2) was first named as Breast Cancer Resistance Protein in the 1990s when it was discovered in MCF-7 breast cancer cell line co-selected for doxorubicin in the presence of verapamil [9]. Following its discovery, ABCG2 was cloned, characterized, and added as the second member of the G subfamily of ABC transporters, as a semi-transport protein [10]. ABCG2 has main effect on effluxing drugs at major physiological barriers, such as blood-brain, blood-testis and maternal-fetal barriers. Similar function of ABCG2 is validated in effluxing of xenobiotics at small intestine and kidney proximal tubule brush borders. ABCG2 transports a wide variety of substrates including several anticancer agents and is one of the most significant contributors to multidrug resistance in cancer cells [10, 11].

Although ABCG2 has been studied in diverse fields, the precise function and effect in RCC are yet poorly understood [12]. Due to the heterogeneous high expression pattern of ABCG2 in the kidney, researchers have speculated that this protein may be actively involved in drug resistance, leading to failure of chemotherapeutic treatments [13, 14]. Clinically, whether ABCG2 expression could predict overall survival for RCC has not been well studied. Thus, we aimed to evaluate the correlation between ABCG2 expression and overall survival of patients with clear cell RCC managed by renal surgery.

## Methods

### Patient samples

Following approval by the Ethics Committee of Ruijin Hospital, the kidney surgery Registry database was used to identify patients who were managed with renal cancer surgery from 2008 to 2013. Written informed consent was obtained from all the patients. During this time, 120 patients with complete recorded information and paraffin sections were enrolled in our study, and followed-up for a minimum of three years. Decisions about radical nephrectomy (114 cases) or partial nephrectomy (6 cases) were made by the primary surgeon based on individual tumor and patient conditions. After surgery, 101 patients without metastasis at the initial diagnosis received interferon alpha for one year as the routine sequential treatment. In addition, Sorafenib or Sunitinib was administered in the other 19 patients with metastatic RCC until intolerable side-effects of drugs or disease progression. The main exclusion criteria from this analysis were a lack of a complete recorded file and unavailability of paraffin sections.

### Evaluation of ABCG2

Immunohistochemistry staining (IHC) is one of the most widely used methods for the identification and assessment of prognostic biomarkers. In the present study, standard IHC protocol was used to detect the expressions of ABCG2 according to the manufacturer’s instructions [14, 15]. Samples of renal cancer were collected, fixed in 10% formalin, and embedded in paraffin wax. Monoclonal antibody against ABCG2 (#271–396, Santa Cruz) was used in this study [14, 15].

Tissue sections were prepared from the formalin-fixed-paraffin-embedded specimens. Antigen retrieval of RCC was performed by incubating the slides in Tris-EDTA buffer (pH 8.4) at 99 °C for 60 min. The endogenous peroxidase activity was inactivated in methanol with 3% H_2_O_2_. Then, the slides were incubated with primary antibody for 60 min and secondary antibody for 8 min, followed by DAB chromagen staining for 8 min. All the procedures were performed using stainer (BenchMark XT, Ventana) and the slides were scanned (Ventana iScan Coreo) [14].

The IHC results were quantified by qualified and experienced pathologists. Each stained section was independently evaluated by two pathologists using standard criteria from the WHO classification. The third independent pathologist would adjudicate the stained section if there was different opinions. The staining of BXP-21 was scored as negative if less than 10% of the tumor cells were stained [14]. The intensity of the positive staining of ABCG2 was graded into two categories: ABCG2(+) for weak membrane staining of tumor cells; ABCG2 (++) for moderate or strong membrane staining of tumor cells.

### TCGA database analysis

The RNA-seq data was downloaded from TCGA database and analyzed by the cBioPortal-MSKCC tool. ABCG2 expression was compared between clear cell RCC and all the other genitourinary tumors available from TCGA database using unpaired *t*-tests and nonparametric test (Additional file 1: Fig. S1). Significance was considered if *p* < 0.05.

### Statistical analysis

Continuous variables were expressed as a median and interquartile range (IQR) and compared using Mann-Whitney test. The categorical variables were compared by chi-square and Fisher’s exact tests. The primary endpoint was defined as the overall survival, which was initiated from the date of renal cancer surgery until death or the end of follow-up. Survival curves were plotted by Kaplan-Meier method and compared using log-rank test. Multivariable Cox regression was used to identify the independent predictors for overall survival. All the *p*-values were two-tailed and *p* < 0.05 was considered as significant. Data were analyzed by using SPSS version 20.0 (SPSS Inc., Chicago, IL, USA).

## Results

### Clinical demographics

We identified 120 patients managed surgically for renal cancer with complete records on analysis, including paraffin sections, surgery type, TNM status parameter, and clinical symptoms at first encounter. All renal cancers were clear cell type and median overall survival of the enrolled subjects was 92.5 (IQR = 62.3–99.4) months. The number of male patients was 83 (69%). The median age was 56 (IQR = 50–65) years, and the median tumor size was 5.0 cm (IQR = 3.5–7.0 cm). During the first diagnosis, 47 (39%) subjects suffered from clinical symptoms including gross hematuria or palpable mass or significant weight loss. Radical nephrectomy was performed on 114 (95%) patients (Table 1).

**Table 1:** Patients and tumor characteristics

Parameters	Value
Number of patients	120
Number of males (%)	83 (70%)
Median age at surgery (years) (IQR)	56 (50–65)
Median follow-up (months) (IQR)	92.5 (62.3–99.4)
Median tumor size (cm) (IQR)	5.0 (3.5–7.0)
Symptoms on first diagnosis (%)	47 (40%)
Tumor stage (TNM2009)	
pT1a	37(30.8%)
pT1b	55 (45.8%)
pT2	18 (15%)
pT3	10 (8.3%)
Regional lymph node metastasis (TNM 2009) (%)	3 (2.5%)
Distant metastasis on first diagnosis (TNM 2009) (%)	19 (16%)
Fuhrman grade	
1	18 (15%)
2	70 (58.3%)
3	25 (20.8%)
4	7 (5.8%)
Type of surgery (%)	
Radical Nephrectomy	114 (95%)
Partial Nephrectomy	6 (5%)
ABCG2 (%)	
(−)	57 (48%)
(+)	52 (43%)
(++)	11 (9.2%)

### Overall survival in the subgroup of ABCG2 expression

**Fig. 1:**
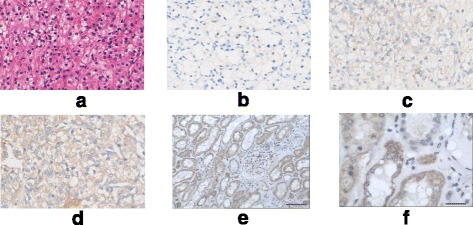
H&E and IHC of the cell membrane in clear cell RCC show the status of ABCG2 (10 × 20). **a** H&E staining of the RCC case. **b** RCC was ABCG2(−), no membrane staining could be seen. **c** RCC was ABCG2(+), weak membrane staining. **d** RCC was ABCG2(++), moderate to strong membrane staining. **e** Scale bar = 50 μm, and **f**. Scale Bar = 200 μm

IHC was used to detect the ABCG2 expression. ABCG2(−) was found in 57 (48%) patients, ABCG2(+) in 52 (43%) patients, and ABCG2(++) in 11 (9.2%) patients (Table 1). IHC-positive staining clear cell RCC cells (brown) were mainly localized on the cell membrane, showing different statuses of ABCG2 (Fig. 1). In order to explore the expression of ABCG2 in non-RCC tissue, we examined another 10 hydronephrosis patients with non-cancer renal tissue and found that ABCG2 was negative in these non-cancer renal parenchyma samples.

**Fig. 2:**
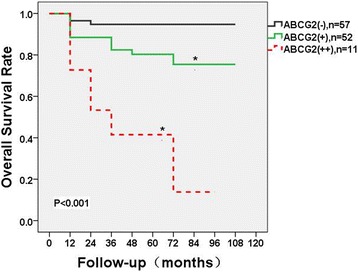
Kaplan-Meier survival for different subgroups based on the expression of ABCG2. A significant difference of overall survival among various degrees of expression of ABCG2 is observed, *p* < 0.001

Kaplan-Meier analysis was used to evaluate overall survival of all patients with clear cell RCC, grouped by ABCG2 expression status. As shown in Fig. 2, median overall survival was 93.2 (IQR = 88.6–96.3) months in ABCG2(−) subgroup, 85.8 (IQR = 40.8–89.9) months in ABCG2(+) subgroup, and 17.0 (IQR = 11.6–48.2) months in ABCG2(++) subgroup. ABCG2 expression significantly associated with the rate of five-year overall survival (*p* < 0.001) (Table 2). The five-year survival rate was 95% in ABCG2(−), 77% in ABCG2(+), and 27% in ABCG2(++) subgroups, respectively (Fig. 2). Besides, there is a significant association between ABCG2 expression and Fuhrman grade (*p* = 0.014). However other clinicopathological parameters, including age, gender, symptoms, pT, lymph node, and surgery type, failed to correlate with ABCG2 expression (Table 2).

**Table 2:** Associations between ABCG2 expression and clinical parameters in patients with clear cell RCC

Parameters	Value	ABCG2(−)	ABCG2(+)	ABCG2(++)	P
Gender	Female	16 (28%)	17 (33%)	4 (36%)	0.510
	Male	41 (72%)	35 (67%)	7 (64%)	
Age at surgery (years)	<60	4172%)	33 (63%)	4 (36%)	0.054
	≥60	16 (28%)	19 (37%)	7 (64%)	
Symptoms on first diagnosis	Negative	36 (63%)	33 (63%)	4 (36%)	0.351
	Positive	21 (37%)	19 (37%)	7 (64%)	
pT	T1a	23 (40%)	13 (25%)	1 (9%)	0.180
	T1b	22 (39%)	28 (54%)	5 (45%)	
	T2	9 (16%)	6 (12%)	3 (27%)	
	T3	3 (5%)	5 (10%)	2 (18%)	
Regional lymph node metastasis	Negative	56 (98%)	51 (98%)	9 (82%)	0.103
	Positive	1 (2%)	1 (2%)	2 (18%)	
Distant metastasis on first diagnosis	Negative	54 (95%)	40 (77%)	7 (64%)	0.001*
	Positive	3 (5%)	12 (23%)	4 (36%)	
Fuhrman grade	1	14 (25%)	4 (8%)	0 (0%)	0.014*
	2	31 (54%)	35 (67%)	4 (36%)	
	3	9 (16%)	11 (21%)	5 (45%)	
	4	3 (5%)	2 (4%)	2 (18%)	
Five-year survival	Alive	53 (93%)	40 (77%)	3 (27%)	<0.001*
	Deceased	4 (7%)	12 (23%)	8 (73%)	
Surgery type	Radical Nephrectomy	2 (4%)	4 (8%)	0 (0%)	0.441
	Partial Nephrectomy	55 (96%)	48 (92%)	11 (100%)	

In multivariable Cox regression analysis, besides Fuhrman grade, ABCG2 expression was another independent factor for overall survival (*p* < 0.001) when incorporating other common clinical parameters, and hazards ratio (HR) was 3.844 (95% CI: 1.919–7.700) (Table 3).

**Table 3:** Multivariable Cox regression for OS in patients with clear cell RCC

Parameters	HR	lower 95% CI	upper 95% CI	P
Gender	1.168	0.469	2.908	0.686
Age at surgery	1.053	0.999	1.110	0.343
Symptoms on first diagnosis	1.610	0.577	4.493	0.468
Clinical tumor size	0.827	0.615	1.111	0.471
pT	2.357	0.432	12.866	0.352
Regional lymph node metastasis	0.404	0.110	1.485	0.220
Distant metastasis on first diagnosis	0.979	0.317	3.019	0.783
ABCG2	3.375	1.704	6.687	0.000*
Fuhrman grade	3.488	1.491	8.161	0.004*

### ABCG2 expression in metastasis cohort

We also found ABCG2 expression significantly associated with distant metastasis on first diagnosis (*p* = 0.001) (Table 2). Patients with metastatic RCC were found during the initial diagnosis, in which 12 patients were presented as ABCG2(+) and 4 as ABCG2(++). The median overall survival was 59.4 (IQR = 43.8–71.3) months and 16.8 (IQR = 14.5–19.8) months, respectively, *p* < 0.001.

### ABCG2 expression in genitourinary tumors from TCGA

Based on the RNA-seq data, the mean value of ABCG2 expression is highest in the clear cell RCC group compared to all the other genitourinary tumors available from TCGA database (Additional file 1: Fig. S1, *p* < 0.001).

The TCGA data about ABCG2 in kidney clear cell cancer are available on the scientific website http://www.oncolnc.org, and http://mexpress.be/. There are significant differences of mRNA levels of ABCG2 between normal kidney tissue and tumor (*p* = <0.0001), and at various stages (*p* = 0.024). Comparison of mRNA levels of ABCG2 were similar in various grades (*p* = 0.503) (Additional file 2: Fig. S2). There is also significant correlation between overall survival and ABCG2 expression (Additional file 3: Fig. S3).

## Discussion

The prognosis of RCC is difficult to predict, and thus, a reliable biomarker is essential to guide the clinical management. RCC is a chemotherapy-resistant and radiotherapy-resistant malignant tumor, and the efficiency of immunotherapy for this type of cancer is also limited [4–6]. The introduction of tyrosine-kinase inhibitors (TKIs) was considered as a breakthrough in the treatment of metastatic RCC [16–18]. However, most metastatic RCC patients administrated with TKIs eventually yield to disease progression due to drug resistance [19]. Several studies have been initiated in an attempt to discover reliable prognostic biomarkers to predict the overall survival of RCC patients. Bui et al. found that carbonic anhydrase IX and Ki67 are useful prognostic biomarkers for RCC that can improve the survival prediction and classification of renal cancer [20]. Several other biomarkers, such as carbonic anhydrase 9, phosphatase, and tensin homologue deleted on chromosome 10, vimentin and p53, are also deemed to correlate with overall survival in RCC patients when in combined clinical parameters [21]. Vermaat et al. also reported that serum amyloid α was a robust and independent prognosticator for overall survival in RCC patients [22].

ABCG2 is a member of the ATP-binding cassette transporters and an ATP-dependent membrane protein predominantly expressed in the kidney [23]. ABCG2 is highly expressed in cancer stem cells or side-population cells and may protect the cells by pumping out xenobiotics, detrimental metabolites of oxidative stress, and chemotherapeutic drugs [23–25]. Recent studies have shown that ABCG2 has a vital role in the multidrug resistance of cancer cells and may influence the overall survival of tumor patients [26, 27]. Yoh et al. reported that in advanced non-small cell lung cancer the response rate to chemotherapy in patients with ABCG2(−) tumors was 44%; however, in ABCG2(+) tumors, this rate decreased to 24%, accompanied by shorter overall survival than ABCG2(−) patients (*p* = 0.004) [28]. Interestingly, by utilizing the resources of TCGA database, we found that the mean expression level of ABCG2 in clear cell RCC is highest compared to all the other genitourinary and gynecologic tumors (Additional file 1: Fig. S1, *p* < 0.001). So we speculate that ABCG2 minght be a crucial marker and regulator of clear cell RCC progression. Therefore, we evaluated the prognostic impact of ABCG2 expression on overall survival in clear cell RCC patients managed with renal surgery.

In the present study, we found that ABCG2 expression highly correlated with overall survival in patients with clear cell RCC. Overall survival is considered as the most valuable endpoint in terms of assessing the prognostic outcome in RCC patients, and the analysis of this parameter is a strength of the study. Survival curves (Fig. 2) showed a significant difference of overall survival among various degrees of expression of ABCG2, *p* < 0.001. The stronger the ABCG2 expression, poorer the overall survival. The median overall survival was 93.2, 85.8, and 17.0 months in ABCG2(−), (+), and (++) subgroups, respectively. The five-year survival rate was 95%, 77%, and 27% in ABCG2(−), (+), and (++) subgroups, respectively. Based on the routine practice in our institution, interferon alpha or TKIs (Sorafenib or Sunitinib) were used as adjuvant therapeutics for patients with RCC after surgery; the efficacy of TKIs was reported as promising [16–18]. However, in our study, 19 patients with metastatic RCC treated with TKIs showed a poor median overall survival.

Moreover, we analyzed the correlation of ABCG2 expression of these 19 patients with metastatic RCC. The median overall survival was 59.4 (IQR = 43.8–71.3) months in ABCG2(+) cohort and 16.8 (IQR = 14.5–19.8) months in ABCG2(++) cohort, *p* < 0.001. The predisposition indicated that stronger the ABCG2 expression, poorer the prognosis. The correlation necessitates further studies. Interestingly, we found that ABCG2 expression significantly correlated with Fuhrman grade in renal cell carcinoma. This may be associated with the protein expression of the nucleus in malignant renal cell. Higher mRNA levels of ABCG2 gene was found in more malignant pancreatic cell [29] and we need to elucidate this in further research in RCC. In the multivariate analysis, ABCG2 and Fuhrman grade were significant predictors for overall survival (*p* < 0.001) when combined with the TNM status parameters.

Due to the limited knowledge and conditions, the mechanism of ABCG2 in RCC is not fully elucidated. Szakacs et al. reported that ABCG2 played a vital role in the multidrug resistance of cancer cells, thereby influencing the overall survival [27]. Hypoxia is the key step in the development and progression of RCC and is mainly regulated by Von Hippel-Lindau, an important tumor suppressor gene. Besides, hypoxia is verified to affect the ATP-binding cassette transporter family, including ABCG2 [30]. In the pancreatic cancer, there are significant correlations between mRNA levels of ABCG2 and clinical outcomes [29]. Many researches are arising to detect the mechanism of ABCG2 in drug resistance in other malignancies, while, to our knowledge, studies regarding ABCG2 expression in RCC leading to therapeutic resistance are lacking. Till date, there is one report by Korenaga and colleagues indicating that the ABCG2 polymorphism (C421A) is a risk factor for developing non-papillary RCC [31]. So it’s necessary to make further study in the field of ABCG2 effect on drug resistance in RCC. With the advent of efficient inhibitors of ABCG2, the combination strategies of targeted drugs and ABCG2 inhibitors might provide the promising therapeutic effect.

The detection ABCG2 expression by IHC staining is clinically valuable. Improved diagnostic techniques aimed at the selection of RCC patients with less expression of ABCG2 might result in more successful outcomes. For example, the RCC patients with highly expressed ABCG2 require more care after surgery and intensive follow-up. Moreover, IHC staining is a common and economical method to detect ABCG2 and could be widely used in clinical management. Therefore, ABCG2 could be utilized in most medical institutions.

While this study comprised a moderate size of patients with extended follow-up, it is also retrospective and derived from a single tertiary-care center, which could impact the generalizability. Another limitation is that the paraffin sections were preserved for a prolonged duration that may affect the IHC staining to a certain degree.

## Conclusions

ABCG2 is a significant and independent prognostic marker of overall survival in patients with clear cell RCC managed by renal surgery, and its high expression is correlated with poor overall survival and increased metastasis. This will be conducive to further research of ABCG2 at molecular level as well as gene level for the drug resistance in kidney cancer. IHC staining for ABCG2 could be monitored routinely in clinical management. Moreover, further pre-clinical evaluations for the mechanism of ABCG2 in RCC are essential.

## Additional files

Additional file 1: Fig. S1. The RNA-seq V2 data was downloaded from TCGA database and analyzed by the cBioPortal-MSKCC tool. Statistical analysis was performed between clear cell RCC and all the other genitourinary tumors available from TCGA database using unpaired *t*-tests and nonparametric test. (Significance was considered as * = *p* < 0.5; **p = < 0.01; *** = *p* < 0.001). UCEC (Uterine Corpus Endometrial Carcinoma) *N* = 177. BUC (Bladder Urothelial Carcinoma) *N* = 408. OSC (Ovarian Serous Cystadenocarcinoma) *N* = 122. KRPCC (Kidney Renal Papillary Cell Carcinoma) *N* = 291. UC (Uterine Carcinosarcoma) *N* = 57. ADC (Adrenocortical Carcinoma (TCGA, Provisional) *N* = 79. KCP (Kidney Chromophobe) *N* = 66. Clear Cell RCC (Kidney Renal Clear Cell Carcinoma) *N* = 598. (PDF 58 kb)

Additional file 2: Fig. S2. The mRNA data was downloaded from TCGA database on the public scientific website: http://mexpress.be/ and drafted into graphics. A, comparison of mRNA levels of ABCG2 between normal kidney tissue and tumor, p = <0.0001. B, comparison of mRNA levels of ABCG2 at different stages, *p* = 0.024. C, comparison of mRNA levels of ABCG2 in different grades, *p* = 0.503. KIRC(Kidney renal clear cell carcinoma). (PDF 381 kb)

Additional file 3: Fig. S3. The TCGA data about ABCG2 expression and overall survival in Kidney clear cancer were available on the website: http://www.oncolnc.org. Kaplan-Meier survival was significant for various groups based on the expression of ABCG2, *p* < 0.001. (PDF 180 kb)

## Abbreviation

ABCG2: ATP-binding cassette sub-family G member 2
ADC: Adrenocortical Carcinoma
BUC: Bladder Urothelial Carcinoma
CI: Confidence Interval
HR: Hazards Ratio
IHC: Immunohistochemistry
IQR: Interquartile Range
KCP: Kidney Chromophobe
KIRC: Kidney Renal Clear cell carcinoma
KRPCC: Kidney Renal Papillary Cell Carcinoma
OSC: Ovarian Serous Cystadenocarcinoma
RCC: Renal Cell Carcinoma
TCGA: The Cancer Genome Atlas
TKIs: Tyrosine-Kinase Inhibitors
UC: Uterine Carcinosarcoma
UCEC: Uterine Corpus Endometrial Carcinoma
