# Supplementary figures and images for: ABCG2 is a potential prognostic marker of overall survival in patients with clear cell renal cell carcinoma
Source: BMC Cancer. 2017 Mar 27;17:222. doi: 10.1186/s12885-017-3224-6 (PMC5368932; doi:10.1186/s12885-017-3224-6)

Supplemental figure 1

ABCG2 Expression RNA-seq

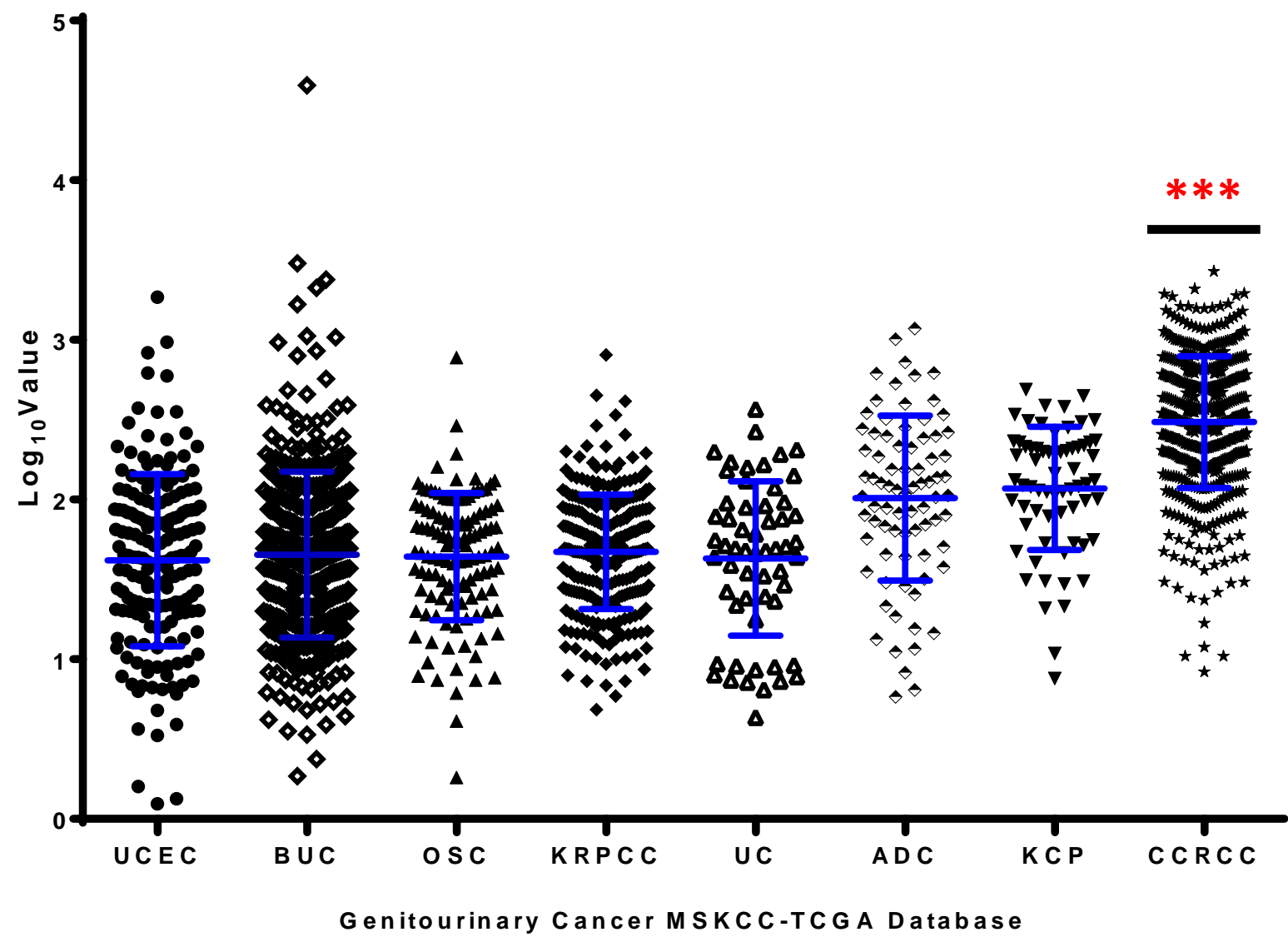

Supplement: Supplementary file 1 — The RNA-seq V2 data was downloaded from TCGA database and analyzed by the cBioPortal-MSKCC tool. Statistical analysis was performed between clear cell RCC and all the other genitourinary tumors available from TCGA database using unpaired t-tests and nonparametric test. (Significance was considered as * = p < 0.5; **p = < 0.01; *** = p < 0.001). UCEC (Uterine Corpus Endometrial Carcinoma) N = 177. BUC (Bladder Urothelial Carcinoma) N = 408. OSC (Ovarian Serous Cystadenocarcinoma) N = 122. KRPCC (Kidney Renal Papillary Cell Carcinoma) N = 291. UC (Uterine Carcinosarcoma) N = 57. ADC (Adrenocortical Carcinoma (TCGA, Provisional) N = 79. KCP (Kidney Chromophobe) N = 66. Clear Cell RCC (Kidney Renal Clear Cell Carcinoma) N = 598. (PDF 58 kb) [file 12885_2017_3224_MOESM1_ESM.pdf]

Supplemental figure 2

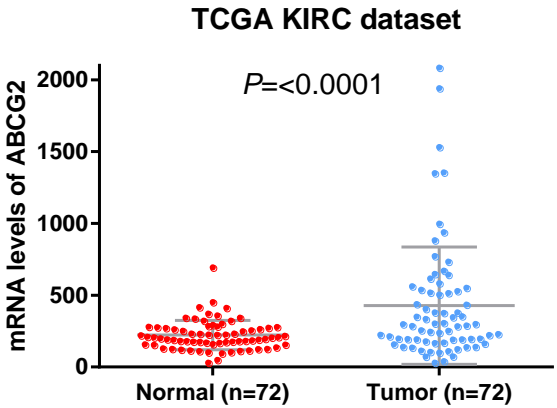

A

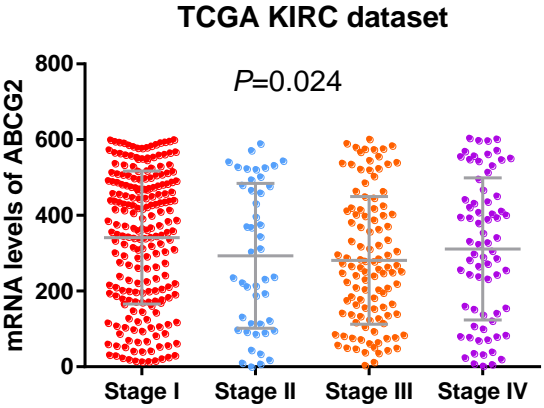

B

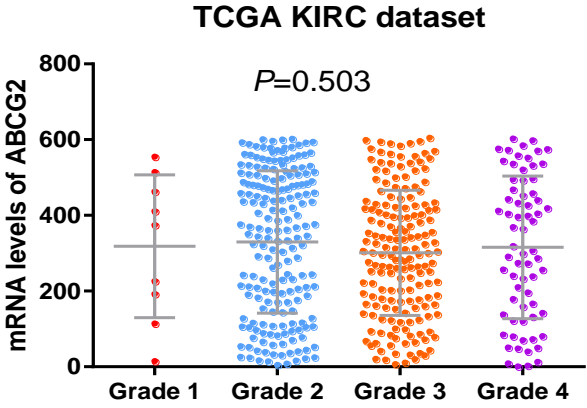

C

Supplement: Supplementary file 2 — The mRNA data was downloaded from TCGA database on the public scientific website: http://mexpress.be/ and drafted into graphics. A, comparison of mRNA levels of ABCG2 between normal kidney tissue and tumor, p = <0.0001. B, comparison of mRNA levels of ABCG2 at different stages, p = 0.024. C, comparison of mRNA levels of ABCG2 in different grades, p = 0.503. KIRC(Kidney renal clear cell carcinoma). (PDF 381 kb) [file 12885_2017_3224_MOESM2_ESM.pdf]

Supplemental figure 3

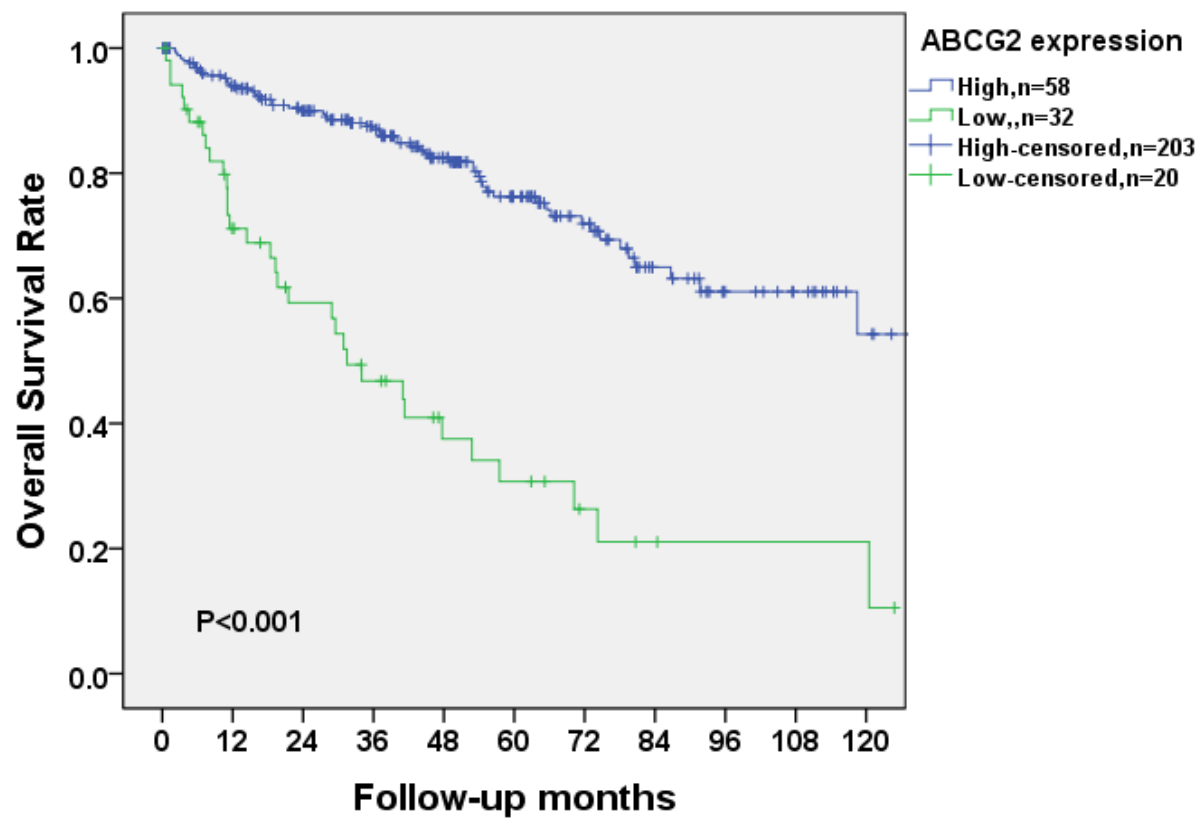

Supplement: Supplementary file 3 — The TCGA data about ABCG2 expression and overall survival in Kidney clear cancer were available on the website: http://www.oncolnc.org. Kaplan-Meier survival was significant for various groups based on the expression of ABCG2, p < 0.001. (PDF 180 kb) [file 12885_2017_3224_MOESM3_ESM.pdf]
